# Investigating the Impact of a Virtual Reality Experience on Medical Student Empathy: Mixed Methods Study

**DOI:** 10.2196/76504

**Published:** 2026-02-04

**Authors:** Allen G Mundok, Vivian N Ho, Lauren A Fowler, Ann Blair Kennedy, Shannon Stark-Taylor

**Affiliations:** 1 Department of Biomedical Sciences, University of South Carolina School of Medicine Greenville, 607 Grove Rd, Greenville, SC, 29605, United States, 1-864-766-2001; 2 Department of Translational Neuroscience, Wake Forest University School of Medicine Charlotte, Charlotte, NC, United States

**Keywords:** medical education, virtual reality, medical students, mixed-methods, empathy

## Abstract

**Background:**

Physician empathy is important not only for improving patient satisfaction and health outcomes but also for increasing physician job satisfaction and protecting against burnout. However, amid concerns over declining empathy levels in medical education, there is a need for innovative teaching approaches that address the empathy gap, a critical element in patient-centered care.

**Objective:**

This study aimed to use a mixed-methods analysis to explore the effectiveness of a virtual reality (VR) intervention versus traditional lecture methods in enhancing empathy among medical students.

**Methods:**

Overall, 50 first- and second-year medical students were randomized to either a VR intervention, which simulated patient experiences, or a control group receiving traditional empathy lectures. Both groups watch 2 videos with reflections gathered after each video to capture students’ experiential learning. Empathy was measured using the Jefferson Scale of Empathy-Student Version before and after the intervention.

**Results:**

Quantitative analysis revealed significant increases in empathy scores post intervention for both groups (lecture group: mean increase 4.71, SD 11.01; VR group: mean increase 5.6, SD 10.02; *P*<.001), indicating that both interventions enhanced empathy. The VR group exhibited a significant difference in qualitative empathy coding after the second video (*U*=165.5; *P*<.001) compared to the lecture group. Qualitative feedback from the VR group emphasized a more profound emotional and cognitive engagement with the patient perspective than the lecture group.

**Conclusions:**

This study supports the integration of VR into medical education as a complementary approach to traditional teaching methods for empathy training. VR immersion provides a valuable platform for students to develop a deeper, more nuanced understanding of empathy. These findings advocate for further exploration into VR’s long-term impact on empathy in clinical practice.

## Introduction 

Empathy can be interpreted as the emotional engagement of the observer with the circumstances of another [1]. In medical settings, higher physician empathy has been associated with improved patient outcomes, including greater patient satisfaction and increased adherence to medical recommendations and treatments [2]. For example, individuals with diabetes whose physicians score higher on measures of empathy demonstrate better glucose and cholesterol control than those whose physicians score lower, controlling for other physician and patient factors [3]. For physicians, empathy has also been linked to higher supervisor ratings of clinical competence and fewer malpractice claims [4,5]. Despite the known benefits of empathy, research also shows that empathy tends to decline during medical school, particularly during clinical training, with male students showing larger decreases [5]. These studies highlight the importance of understanding how empathy develops and changes throughout the course of one’s medical education and suggest a need for interventions to support its maintenance.

Studies have demonstrated the effectiveness of many different modes of empathy training for increasing empathy ratings in medical students [6,7]. To train individuals in empathy, traditional interventions have often taken the form of perspective-taking. These interventions involve having participants imagine another’s situation and put themselves “in that person’s shoes.” Perspective-taking has also been shown to promote empathy in a range of nonmedical contexts [8-10].

Compared to traditional perspective-taking, virtual reality (VR) interventions offer a fully immersive experience, giving the user the visual and emotional feeling of being present for the interaction and embodying another [11,12]. One study compared traditional perspective-taking to a VR perspective-taking intervention using a virtual environment to promote increased empathy for homeless persons. Participants who underwent the VR intervention showed more positive, longer-lasting attitudes toward the homeless and signed a petition supporting the homeless at a significantly higher rate than those who engaged in a traditional perspective-taking task [11]. Studies also suggest VR interventions are less cognitively taxing than imagining another’s perspective and allow for improved methodology as all participants engage in the same exercise [13-15].

Despite this evidence, few studies have applied VR technology to teaching medical students and physicians. With medical students increasingly using technology resources to enhance their classroom learning [16,17], finding ways to integrate technology into empathy training may be an effective method to engage medical students [18,19]. Given the benefits of immersive perspective-taking in other contexts, we designed an innovative VR experience to facilitate patient perspective-taking among medical students and compared it to traditional teaching methods (a video lecture). Based on prior findings promoting empathy for several different human conditions in lay populations and the theoretical advantages of VR for perspective-taking, we hypothesize that engaging in patient perspective-taking through VR will result in greater increases in empathy among medical students than the traditional teaching method.

## Methods

### Study Design and Video Development

To compare the efficacy of a VR experience to traditional lecture learning, we used a randomized controlled mixed-methods convergent parallel design. In this study design, both qualitative (structured reflection responses) and quantitative data (Jefferson Scale of Empathy for medical students [JSE-S] pre- and posttest scores) were collected simultaneously in a single phase. Each type of data were analyzed separately, then merged to identify points of convergence [20]. This design allows us to integrate the strengths of both approaches, providing a more comprehensive understanding of how the VR intervention influenced empathy, while also allowing the qualitative data to contextualize and enrich the quantitative findings [20].

The lecture videos were PowerPoint lectures designed and narrated by a biomedical sciences professor and research team member (LAF) with expertise in empathy. Lecture video 1 (V1) described the science behind empathy and its importance in health care. Lecture video 2 (V2) provided strategies for students to enhance empathy in clinical practice.

To make the VR videos and reflection questions as authentic as possible, these items were presented to a panel of patients and providers trained in giving feedback to researchers. The panel members made suggestions for script and question improvements. After incorporating the received feedback into the revised script, the VR videos were filmed using a Garmin VIRB Ultra 360 camera (Garmin, Ltd.) with hired actors playing the roles of a patient and physician. VR Video 1 presented a third-person perspective of a “normal” patient-physician interaction during a routine office visit. VR Video 2 showed the same interaction but through the first-person perspective of the patient, and the patient’s inner thoughts were projected as text on the screen for students to read. An example of the patient’s thoughts is, “How am I going to afford a dietician? Will my insurance pay for that?” Videos for the lecture and VR group were designed to be the same length to control for time.

### Data Collection

**Figure 1.**
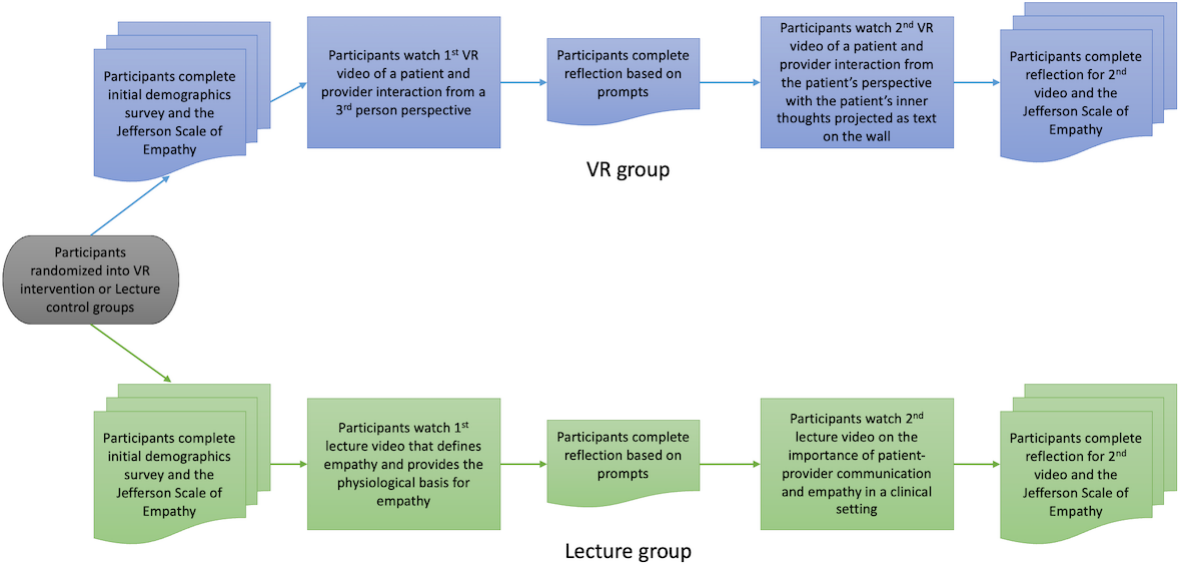
Study design overview. Flowchart depicting participant randomization into virtual reality or lecture video group and timeline of assessment (demographics survey, Jefferson Scale of Empathy pre- and postvideos, and reflection prompts). VR: virtual reality.

Using a random number generator, students were randomly assigned to either the lecture group (n=25) or VR group (n=25). In the lecture group, students were given a tablet to watch the 2 PowerPoint lecture videos on empathy. In the VR group, students wore a VR headset to watch the 2 VR videos depicting a patient-physician interaction. Prior to watching their respective videos, students completed an initial demographics survey and the Jefferson Scale of Empathy for medical students (JSE-S). After watching the first video, students answered 3 open-ended reflection questions on their learning experience. Students then watched the second video and again completed the 3 reflection prompts and the JSE-S. Figure 1 depicts this methodology.

Throughout their viewing of the videos, students in both groups were also equipped with Empatica E4 wristbands (Empatica, Inc.) to measure physiological markers of empathy such as galvanic skin response and heart rate variability. While physiological markers have been used in prior research to complement self-reported empathy measures, technical artifacts in our dataset rendered the recordings uninterpretable; these data were therefore not included in our final results [21].

### Instruments

The validity of the JSE in evaluating empathy in physicians and medical students has been well-established [22,23]. We used the student version (JSE-S), which was developed to measure medical students’ attitudes toward empathy in patient-physician interactions. The JSE comprises 20 items, rated on a 7-point Likert-type scale (1=strongly disagree, 7=strongly agree), with higher total scores indicating greater empathy (maximum score=140). An example of a typical item is, “Patients feel better when their health care providers understand their feelings.” The JSE-S has demonstrated strong internal consistency, construct validity, and predictive associations with clinical competence and patient-centered outcomes [24,25]. Although the JSE-S primarily assesses attitudes toward empathy rather than direct empathic behavior, it is widely accepted as a valid proxy for empathy in medical education research [26]. Empathy in clinical contexts is understood as a multidimensional construct encompassing cognitive, affective, and attitudinal components [22]. The attitudinal dimension captured by the JSE-S reflects physicians’ and students’ willingness and value orientation toward understanding patients’ experiences, which are key determinants of empathetic engagement in practice. Thus, while the JSE-S measures empathy-related attitudes, its theoretical foundation and empirical validation support its continued use as a reliable instrument for assessing empathy development in medical students.

To complement the JSE-S and provide qualitative insight into students’ experiences, participants also responded to structured reflection questions. These served as our qualitative data collection tool, analogous to an interview guide. The lecture group questions asked students to describe the video, to describe how the video made them feel, and to explain how they would apply the information in future practice. The VR group questions asked students to describe the video, to describe how the video made them feel, and to explain how they thought the patient felt during the interaction. While these reflections are self-reported and may be influenced by social desirability or awareness of study aims, they provide rich, contextual data that complement the quantitative JSE-S measures.

### Statistical Analysis

We conducted qualitative analysis based on the participants’ responses to the reflection prompts using a thematic analysis approach as outlined by Braun and Clarke [27-29]. Written responses were imported into Microsoft Excel for data management. Given the dataset in this study was relatively small (n=50 participants, 100 reflections), Microsoft Excel was sufficient for systematically organizing codes and themes, while analytic rigor was ensured through team-based coding and consensus review. Initial codes were created by reading the text provided by the participants and assigning a code identifier. Coding was an iterative process in which each instance of a code was compared to previous instances to confirm or modify the code and its definition. Once all the data were coded, emergent themes were abstracted by grouping similar codes together. Codes and themes were each reviewed by members of the research team (AM, VH, ABK, and SST). Any discrepancies were resolved through discussion until consensus was reached. This process represents a “modified” application of thematic analysis, and codes were later used to derive quantitative empathy scores as described below. Major themes and illustrative quotes from the participants’ responses are reported in the results section to provide further context and to support our findings.

We then determined quantitative scores based on the level of empathy present in the participants’ responses. We assigned empathy scores to each participant based on their responses to video 1 (V1) and responses to video 2 (V2), and we also assigned an overall empathy score based on their responses to both videos 1 and 2 (V3). Empathy was scored on a 3-point scale (1=no signs of empathy, 2=some signs of empathy, 3=high signs of empathy). We used our codes to assign these empathy scores. High signs of empathy were indicated by codes such as “patient focused” and “emotive words,” while low signs of empathy were indicated by codes like “surface level” and “misperception.” We also used a 3-point scale to assign each participant a score for level of change in assigned empathy scores from video 1 to video 2 (1=negative change, 2=no change, and 3=positive change).

The data were quantitatively analyzed by LAF using IBM SPSS Statistics (version 29.0). Descriptive statistics (counts and frequencies for categorical data; means and SD values for continuous data) were performed. We used Mann-Whitney tests to compare the assigned empathy scores at V1, V2, and V3 between the lecture and VR groups. JSE-S scores pre- and posttest were analyzed using paired-sample 2-tailed *t* tests to determine whether JSE-S scores changed over time for all participants. A factorial analysis of variance assessed whether time (pre or posttest) and group (lecture or VR) affected JSE-S scores. To confirm a relationship between the JSE-S and our assigned empathy scores, we ran a Spearman correlation between posttest JSE-S score and V3 for both lecture and VR groups. We considered *P*<.05 as statistically significant.

### Ethical Considerations

From January to February of 2020, 50 first- and second-year medical students at the University of South Carolina School of Medicine Greenville participated in our study. The study was reviewed and approved by the University of South Carolina Office of Research Compliance (Institutional Review Board approval number: Pro00089391). Participation was voluntary, and students received a $30 gift card as compensation. All participants provided written informed consent prior to participation. Upon arrival, study personnel explained the study procedures and reviewed the informed consent document with each participant, answering all questions before consent was obtained. To minimize response bias, recruitment materials and the consent process described the study as an investigation of the role of virtual reality in medical education, which was accurate but did not fully disclose that empathy was a primary outcome of interest. Participants were verbally debriefed immediately following completion of their participation and informed of the study’s full purpose. The study posed minimal risk to participants. Potential risks included mild skin irritation from the polyurethane E4 wristband and nausea or headache associated with use of the virtual reality headset. Participants were informed they could discontinue use of either device or withdraw from the study at any time without penalty. Identifying information was collected solely for recruitment purposes and was not linked to study data. Recruitment information was stored on a password-protected Microsoft Teams site accessible only to study personnel. All study data were deidentified, assigned a unique participant identification number, and stored in a HIPAA-compliant REDCap database on a secure network accessible only to authorized members of the research team.

## Results

### Participant Characteristics

Of the 50 preclinical medical students that participated in our study, the overall mean age was 24.14 (SD 2.66) years. The majority of participants were female (37/50, 74%) and non-Hispanic White (31/50, 62%). There were similar numbers of participants in each class year, with 24 of 50 (48%) M1 students and 26 of 50 (52%) M2 students. Eight of 50 (16%) participants identified as first-generation students, and 14 of 50 (28%) participants were from rural backgrounds. The specialty of interest varied among the participants, but the highest number of students (11/50, 22%) chose obstetrics and gynecology. Additional demographic information is depicted in Table 1.

**Table 1.** Participant demographic data.

Variables		Lecture, (n=25)	VR^a^, (n=25)	Total, (N=50)
Age (years), mean (SD)		23.92 (1.53)	24.36 (3.46)	24.14 (2.66)
Sex, n (%)				
Male		8 (32)	4 (16)	12 (24)
Female		16 (64)	21 (84)	37 (74)
Transgender		1 (4)	0 (0)	1 (2)
Race and ethnicity^b^, n (%)				
Asian		2 (8)	7 (28)	9 (18)
Black or African American		4 (16)	5 (20)	9 (18)
Hispanic or Latino		1 (4)	0 (0)	1 (2)
Non-Hispanic White		18 (72)	13 (52)	31 (62)
Another or not listed		2 (7.7)	0 (0)	2 (4)
Decline to answer		0 (0)	1 (4)	1 (2)
Class year, n (%)				
M1		13 (52)	11 (44)	24 (48)
M2		12 (48)	14 (56)	26 (52)
Marital status, n (%)				
Single or never married		23 (92)	21 (84)	44 (88)
Married		2 (8)	4 (16)	6 (12)
First generation^c^, n (%)		4 (16)	4 (16)	8 (16)
Rural, n (%)		7 (28)	7 (28)	14 (28)
Specialty, n (%)				
Dermatology		0 (0)	2 (8)	2 (4)
Emergency medicine		2 (8)	1 (4)	3 (6)
Family medicine		1 (4)	2 (8)	3 (6)
General surgery		6 (24)	2 (8)	8 (16)
Internal medicine		2 (8)	1 (4)	3 (6)
Internal medicine-pediatrics		1 (4)	0 (0)	1 (2)
Interventional radiology		1 (4)	0 (0)	1 (2)
Obstetrics and Gynecology		5 (20)	6 (24)	11 (22)
Ophthalmology		1 (4)	0 (0)	1 (2)
Orthopedic surgery		2 (8)	2 (8)	4 (8)
Otolaryngology		1 (4)	1 (4)	2 (4)
Pediatrics		1 (4)	5 (20)	6 (12)
Undecided		2 (8)	3 (12)	5 (10)

a VR: virtual reality.

b Response options allowed respondents to check more than 1 option.

c In our study, defined as a student whose parents or guardians did not complete a four-year degree.

### Quantitative Analysis: JSE-S

**Figure 2.**
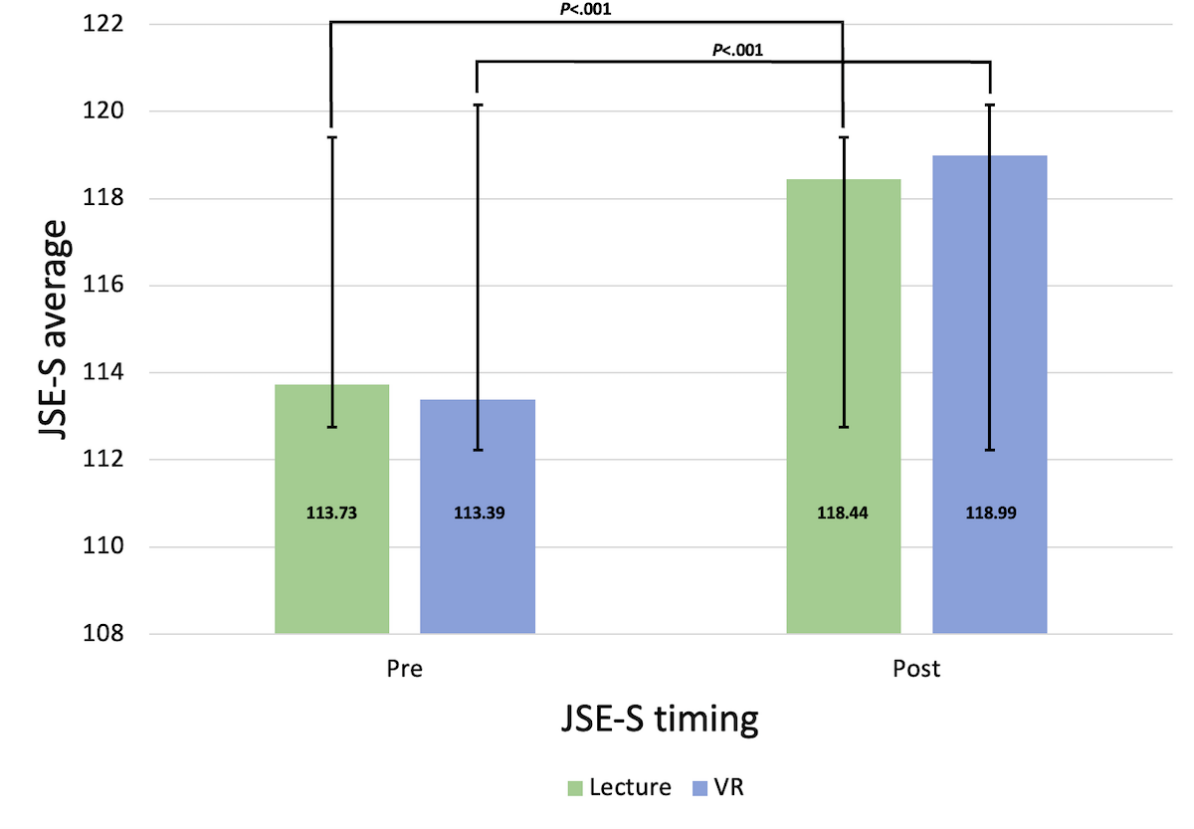
Average scores across time (pre or post) by group (lecture or VR). The Jefferson Scale of Empathy-Student Version was administered to participants prior to viewing both videos (pre) and after viewing both videos (post). Jefferson Scale of Empathy-Student Version score was compared across time in lecture versus VR groups using a paired-sample *t* test (***P<.001). SD bars are shown. JSE-S: Jefferson Scale of Empathy-Student Version; VR: virtual reality.

JSE-S scores were assessed prior to and following each testing session (pre or posttest) for both the lecture and VR groups. Prevideo JSE-S scores were not significantly different between the groups, with the lecture group averaging 113.73 (SD 10.57) and the VR group averaging 113.39 (SD 10.24). Postvideo averages of the JSE scores between the 2 groups were again not significantly different, showing that the lecture group averaged 118.44 (SD 11.01) and the VR group averaged 118.99 (SD 10.02). A paired-samples *t* test was used to determine whether JSE-S scores changed across time for all participants. Results indicate that JSE scores significantly increased after the empathy training sessions compared to before the sessions (*t*_49_=7.28; *P*<.001). A factorial analysis of variance assessed whether time (pre or post) and group (lecture or VR) affected JSE-S scores, with results indicating no significant effect of either (*F*_1,48_=0.17; *P*=.68). These results indicate that while there was no statistically significant difference in JSE-S scores observed between the lecture and VR groups, both groups were effective in significantly increasing empathy scores. Refer to Figure 2 for comparison of JSE-S scores across time for both groups.

### Qualitative Analysis of Reflection Responses

**Figure 3.**
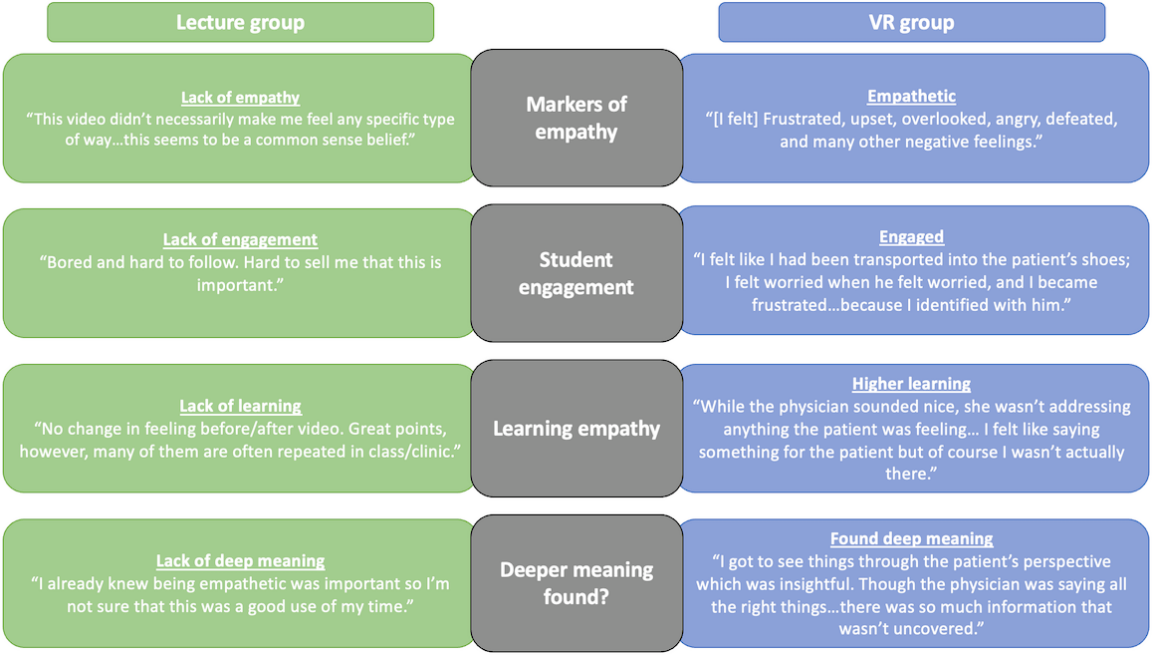
Major themes and representative quotations. Qualitative coding of participant reflection responses revealed 4 major themes. Example quotations representative of each theme are shown to compare lecture versus VR groups. VR: virtual reality.

Qualitative analysis of participants’ reflection responses in the lecture group versus the VR group revealed 4 major themes relating to empathy and its learning that diametrically opposed each other: (1) markers of empathy, (2) student engagement, (3) learning empathy, and (4) deeper meaning found (Figure 3). The lecture group responses overall demonstrated lack of empathy, lack of engagement, lack of learning, and lack of deeper meaning. While lecture group students seemed to cognitively acknowledge the importance of empathy, their responses were generally sarcastic and demonstrated low levels of empathy. For example, when asked how the lecture video made them feel, 1 student responded simply with, “bored.” Another stated, “this video made me feel sleepy…this is all stuff we have heard before.” When asked how they would apply the lecture content to future clinical practice, 1 student responded with “be empathetic like I was supposed to be before?” In contrast, the VR group responses showed empathy, engagement, higher learning, and deeper meaning. VR group students experienced a range of empathetic emotions after the VR experience, with 1 student stating that they felt “frustrated, upset, overlooked, angry, defeated, and many other negative feelings” on behalf of the patient. The VR group participants were highly engaged, commenting, “I felt like stepping in and asking the patient for input,” and “I felt like I wanted to be Sam’s advocate and ask questions…the provider should’ve asked.” Because the VR videos promoted perspective-taking, the students found deeper meaning. One individual stated, “I think the patient…needed to talk about his feelings just as much, if not more than his abdominal pain.” The VR group also demonstrated learned empathy in their responses. After watching the first video, 1 participant commented, “I felt that the physician was doing a good job being pleasant.” After the second video, the same participant stated, "I felt like I had been transported into the patient’s shoes; I felt worried when he felt worried, and I became frustrated when the doctor didn’t give him any feasible solutions because I identified with him at that point.”

### Quantitative Scoring of Qualitative Coding and Comparison to JSE-S Scores

**Figure 4.**
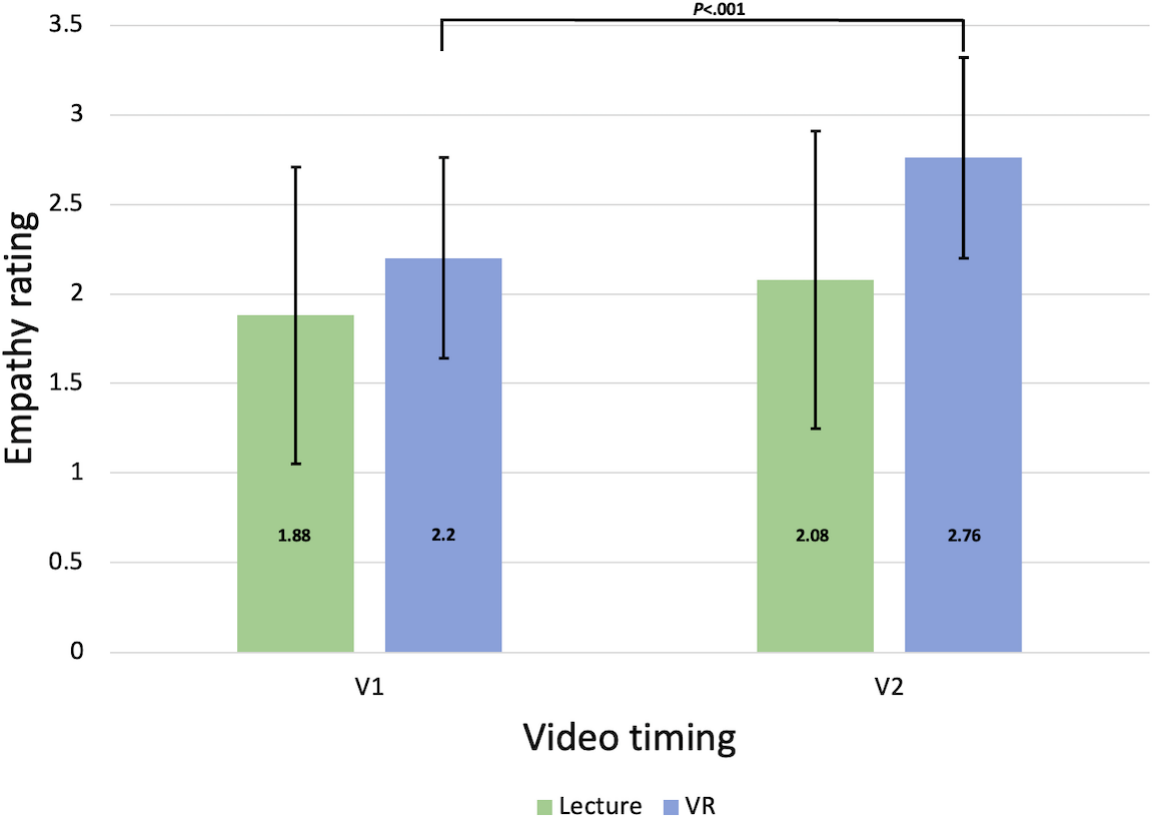
Qualitative scoring of comments after video 1 (V1) and video 2 (V2). V1 is defined as participants’ assigned empathy score after viewing Video 1. V2 is defined as participants’ assigned empathy score after viewing Video 2. A Mann-Whitney analysis compared the assigned empathy scores between V1 and V2 in the lecture and VR groups (***P<.001). SD error bars are shown. VR: virtual reality.

A Mann-Whitney analysis was used to compare the assigned empathy scores at V1 (after video 1) and V2 (after video 2) between the lecture and VR groups. Results indicate that there was no significant difference in qualitative coding for empathy scores between the 2 groups after the first video (V1). However, after the second video (V2), there was a significant difference between qualitative scores, with those in the VR group having significantly higher empathy coding scores than the lecture group (*U*=165.5; *P*<.001). A Mann-Whitney analysis also showed that overall qualitative empathy coding for the participants (V3) was significantly higher for those in the VR group as compared to those in the lecture group (*U*=202.5; *P*=.03), based on empathy scores of 1.96 and 2.52 in the lecture and VR groups, respectively.. The overall qualitative coding for empathy (V3) was assessed with the post-JSE-S scores to determine if the 2 scores were related to each other (a quantitative rating of empathy in the JSE-S compared to the quantitative scoring of the qualitative coding). Spearman correlation between posttest JSE-S and V3 for both the lecture and VR groups was significantly, positively correlated (ρ_49_=0.29; *P*=.03). Refer to Figure 4 for more information.

## Discussion

### Principal Findings

This study presents early findings that the VR experience uniquely engaged students and elicited more empathetic responses compared to the traditional lecture. In our mixed methods evaluation, the intervention or VR group demonstrated qualitatively higher markers of empathy, more student engagement, found deeper meaning, and demonstrated more learning of empathy than the lecture group. The intervention or VR group also showed significantly higher overall assigned empathy scores than the lecture group. These findings indicate that immersive VR experiences may enhance empathy development beyond conventional lecture-based methods.

### Quantitative and Qualitative Findings

The JSE-S is widely used and has been validated by numerous empirical studies as an instrument to measure empathy [22,30]. Our quantitative analysis conducted using this tool demonstrated a significant increase in JSE-S scores after both the VR and traditional lecture training sessions compared to before the sessions. Seeing an increase in JSE-S scores in both groups posttest is not a surprising result considering students were primed on the topic of physician empathy while completing the questionnaire and since traditional teaching methods have already been proven effective in teaching empathy to some degree [6,31,32]. However, the VR intervention’s efficacy in fostering empathy demonstrates noninferiority to traditional lecture-based learning and underscores the importance of experiential learning, as outlined in adult learning theory, and provides a new dimension to empathy theory in medical education. Adult learning theory emphasizes the significance of self-directed, experiential learning processes, where adults learn best when engaged in activities that relate closely to real-life scenarios or challenges they might face [33]. The immersive nature of VR, by providing medical students with a first-person perspective of patient experiences, leverages these principles effectively, offering a profound, emotive learning experience that traditional didactic methods may lack. This approach aligns with the constructs of empathy theory, which posits that empathy involves not just cognitive understanding but also affective sharing and emotional resonance with others [34].

Qualitative analysis helped us better understand the experiential differences between the groups. Themes from the qualitative responses relate clearly to the literature on empathy and adult learning. Individuals in the VR group described experiencing a variety of emotions while watching the VR video, while the lecture group described their experience using more cognitive language. This suggests that the VR intervention met our intent to promote perspective taking. Furthermore, adult learning theorists emphasize the role emotions can play in helping learners connect a construct with prior knowledge and past experiences [35]. Beyond enriching their learning through perspective taking, the VR intervention appeared to uniquely engage students more than the lecture. The theme of deeper meaning found in the VR group’s reflections aligns with the concept of “presence” in VR environments, the true feeling of “being there” in the virtual space. This sense of presence is critical for generating impactful, empathetic responses, as it bridges the gap between knowing about another’s experience and feeling with them. Literature on VR and empathy underscores the role of presence in enhancing the emotional impact of VR experiences, suggesting that it is this immersion that enables learners to connect with content on a deeper emotional level, fostering a genuine understanding and appreciation for the patient’s perspective [36].

By connecting the quantitative increases in JSE-S scores with the rich qualitative reflections, a clear pattern emerges: immersive VR experiences not only improve self-reported empathy but also foster deeper emotional engagement, perspective-taking, and understanding of patient experiences. This combined evidence highlights the main contribution of the study, showing that VR interventions can meaningfully complement and enhance traditional teaching methods.

### Implications for Medical Education

For this topic in particular, embodied and applied experience seems to provide much more nuance to the concept of empathy versus the cognitive acknowledgment that empathy is important in practice. Through the comments, the intervention appeared to help many students more fully understand how empathy can promote better health care. Furthermore, this study’s findings resonate with previous research indicating the potential of VR to overcome some of the limitations associated with traditional empathy training methods. For instance, studies have demonstrated VR’s effectiveness in reducing cognitive load and allowing for a more standardized and immersive learning experience, characteristics that are particularly advantageous in the context of medical training where the cognitive demand is high and the need for consistent, reproducible training experiences is paramount [11].

### Limitations and Future Directions

Our review consisted of a single-institution study at 1 academic center, meaning more work is needed to make more widespread application of our results possible. We note that our participants were predominantly female (37/50, 74%). This reflects the voluntary nature of our participant recruitment, as a greater number of female students elected to enroll compared to their male counterparts. Prior literature has documented sex and gender differences in empathy [37]; thus, we acknowledge the overrepresentation of female participants in our study as a factor that may have influenced the observed results. Furthermore, we did not follow up with participants after the end of the study to assess empathy during clinical rotations and in practice. We feel this would have provided a better understanding of the long-term impact of our empathy training intervention on participants’ empathy throughout their medical training. We also recognize some limitations in our use of the JSE-S, including the fact that we may have applied the scale too early following our intervention, thus potentially limiting its reliability. Compared to other interventions to promote empathy in health care learners, this intervention was quite brief; a systematic review of such interventions found that the average length was 10 hours [38].

While traditional teaching methods have been shown to help increase empathy in medical students, the profound differences noted in our qualitative data, however, lead us to question whether full immersion through a VR experience may be a helpful additional tool in allowing students to develop a deeper understanding. Future directions warrant testing a combined approach of traditional lecture alongside a VR intervention to see if a combination provides an even greater increase in empathy than either one alone. We would also like to assess the long-term impact of these interventions on students as they progress through their clinical years.

Although the JSE-S is widely used and validated, it primarily assesses attitudes toward empathy rather than direct empathetic behavior, and participants may respond in a socially desirable manner. No filler items were included to mask the study’s purpose, but participants were not explicitly informed of the study hypotheses to minimize expectance effects. We acknowledge the Hawthorne effect, in which participants modify behavior because they know they are being observed, could have influenced responses. However, the randomized controlled design, balanced group assignments, and inclusion of both qualitative and quantitative data help mitigate potential bias and strengthen the validity of the findings. Future studies might incorporate validated infrequency or social desirability scales, such as the Infrequency Scale from the Zuckerman-Kuhlman Personality Questionnaire to further account for response bias.

### Conclusions

Our study underscores the transformative potential of VR interventions in enhancing empathy among medical students. By allowing learners to experience the patient’s perspective firsthand, VR offers an emotionally engaging, experiential form of learning that complements traditional didactic methods. Our findings, demonstrating higher JSE-S scores and richer qualitative reflections in the VR group, indicate that immersive learning environments can promote deeper perspective taking and maintain empathic growth. These findings illuminate the importance of adopting innovative, technology-enhanced educational strategies to cultivate a more empathetic health care workforce. They also suggest that technology-enhanced approaches may help counter the well-documented decline in empathy during medical training. Integrating VR into medical curricula could provide a scalable, standardized way to strengthen the humanistic foundations of physician education while aligning with contemporary adult learning principles. Future research should examine the long-term effects of such interventions and explore combinations of VR with reflective or clinical teaching to reinforce empathetic skills in practice. Our work lays a foundational step toward reimagining empathy training in medical education by leveraging immersive technology to cultivate emotional understanding and advance a more holistic model of physician training that integrates scientific expertise with the empathy essential to compassionate, patient-centered care.

## Abbreviations

JSE-S: Jefferson Scale of Empathy for medical students
V1: lecture video 1
V2: lecture video 2
V3: lecture video 3
VR: virtual reality
